# Dietary Fat Alters Body Composition, Mammary Development, and Cytochrome P450 Induction after Maternal TCDD Exposure in DBA/2J Mice with Low-Responsive Aryl Hydrocarbon Receptors

**DOI:** 10.1289/ehp.0800530

**Published:** 2009-05-18

**Authors:** Michele La Merrill, Bittu S. Kuruvilla, Daniel Pomp, Linda S. Birnbaum, David W. Threadgill

**Affiliations:** 1 Department of Genetics, Curriculum in Toxicology, Center for Environmental Health and Susceptibility, Clinical Nutrition Research Unit, Lineberger Cancer Center and Carolina Genome Sciences Center, University of North Carolina at Chapel Hill, Chapel Hill, North Carolina, USA; 2 Experimental Toxicology Division, U.S. Environmental Protection Agency, Office of Research and Development/National Health and Environmental Effects Research Laboratory, Research Triangle Park, North Carolina, USA; 3 Department of Genetics, North Carolina State University, Raleigh, North Carolina, USA

**Keywords:** aryl hydrocarbon receptor, fetal loss, gene-environment interactions, mouse, obesity, 2,3,7,8-tetrachlorodibenzo-*p*-dioxin (TCDD)

## Abstract

**Background:**

Increased fat intake is associated with obesity and may make obese individuals uniquely susceptible to the effects of lipophilic aryl hydrocarbon receptor (AHR) ligands.

**Objectives:**

We investigated the consequences of high-fat diet (HFD) and AHR ligands on body composition, mammary development, and hepatic P450 expression.

**Methods:**

Pregnant C57BL/6J (B6) and DBA/2J (D2) dams, respectively expressing high- or low-responsive AHR, were dosed at mid-gestation with TCDD. At parturition, mice were placed on an HFD or a low-fat diet (LFD). Body fat of progeny was measured before dosing with 7,12-dimethylbenz[*a*]anthracene (DMBA). Fasting blood glucose was measured, and liver and mammary glands were analyzed.

**Results:**

Maternal TCDD exposure resulted in reduced litter size in D2 mice and, on HFD, reduced postpartum survival in B6 mice. In D2 mice, HFD increased body mass and fat in off-spring, induced precocious mammary gland development, and increased AHR expression compared with mice given an LFD. Maternal TCDD exposure increased hepatic *Cyp1a1* and *Cyp1b1* expression in offspring on both diets, but DMBA depressed *Cyp1b1* expression only in mice fed an HFD. In D2 progeny, TCDD exposure decreased mammary terminal end bud size, and DMBA exposure decreased the number of terminal end buds. Only in D2 progeny fed HFD did perinatal TCDD increase blood glucose and the size of mammary fat pads, while decreasing both branch elongation and the number of terminal end buds.

**Conclusions:**

We conclude that despite having a low-responsive AHR, D2 progeny fed a diet similar to that consumed by most people are susceptible to TCDD and DMBA exposure effects blood glucose levels, mammary differentiation, and hepatic *Cyp1* expression.

The environmental pollutant 2,3,7,8-tetra-chloro dibenzo-*p*-dioxin (TCDD) is implicated in a variety of metabolic and endocrine toxicities ranging from body weight changes and type II diabetes to altered puberty and breast cancer risk (Brown et al. 1998; Fujiyoshi et al. 2006; Zhu et al. 2008). Much of this toxicity is ascribed to the nuclear receptor activity of TCDD. Polyhalogenated aromatic hydrocarbons (PHAHs) and polycyclic aromatic hydrocarbons (PAHs), such as TCDD and 7,12-dimethylbenz[*a*]anthracene (DMBA), respectively, bind the aryl hydrocarbon receptor (AHR) to induce cytochrome P450, polypeptide 1 (CYP1) enzymes (Nebert et al. 2004; Postlind et al. 1993). The differential activity of *Ahr* alleles influences the level of CYP1 induction and the sensitivity to many toxicities. Induction of CYP1A1 by TCDD is approximately 10 times lower when the *Ahr^d^* allele is activated compared with the *Ahr^b^* allele (Poland and Glover 1980). These allelic differences likely correlate to structural differences that cause lower ligand affinity of the AHR^d^ peptide (Chang et al. 1993). When AHR^d^ is bound by less potent ligands, such as DMBA, no CYP1A1 induction occurs, and levels of CYP1B1 are lower than found in the presence of AHR^b^ (Galvan et al. 2003, 2005). It is these allelic differences in *Ahr* that are believed to underlie the differential sensitivity of C57BL/6J (B6) mice (*Ahr^b1^* allele) to TCDD and DMBA toxicities compared with DBA/2J (D2) mice (*Ahr^d^* allele), which are resistant to TCDD toxicity and lack a DMBA response (Sato and Tomita 1998; Weber et al. 1995). Although much of the research on AHR ligands has been conducted in B6 mice, evidence from CYP1A1 induction suggests that the *Ahr^d^* allele of D2 is more comparable to the *Ahr* allele of humans (Moriguchi et al. 2003).

Because TCDD is lipophilic and persistent, the rise of obesity prevalence may lead to an increased risk of TCDD exposure. Diets that contribute to obesity, such as consumption of high-fat, animal-based foods, are positively correlated with human serum and milk levels of dioxins (Goldman et al. 2000; LaKind et al. 2004). Consequently, increased food intake, particularly from a high-fat diet (HFD), may also increase risk of dioxin toxicities through higher intakes of TCDD. Furthermore, elimination of TCDD is slowed significantly with increasing adiposity (Michalek and Tripathi 1999) because of a larger adipose volume for TCDD distribution (Emond et al. 2006). This effect has been recapitulated in B6 mice, which when fed an HFD have a TCDD elimination half-life 2.4 and 1.4 times longer in liver and adipose, respectively, compared with those on a normal diet (DeVito et al. 2003). However, it is unclear whether obese individuals that are AHR ligand resistant, like D2 mice (Moriguchi et al. 2003), are susceptible to lipophilic AHR ligand toxicities.

Endocrine disruption by dietary fat and obesity, or exposure to AHR ligands can alter mammary gland development during puberty. Dietary fat induces early breast development (Britton et al. 2004), and girls with higher body fat have earlier breast development than do their lean counterparts (Carmichael 2006; Himes et al. 2004; Ogden et al. 2006). Conversely, as serum dioxin concentrations increase in girls, their pubertal breast development is delayed (Den Hond et al. 2002).

In this study, we sought to examine how dietary fat modifies the influence of AHR ligands on pubertal body composition and P450 expression. To account for the role of AHR ligand affinity, in this study we evaluated female B6 mice, which express high- affinity AHR^b1^, making them sensitive to AHR ligands, and D2 mice, which express low-affinity AHR^d^, making them less sensitive to TCDD and nonresponsive to DMBA induction (Chapman and Schiller 1985; Moriguchi et al. 2003). We determined the effects of maternal TCDD exposure and dietary fat on survival, body mass, body fat composition, and fasting glucose levels in offspring. We further addressed to what extent dietary fat and developmental TCDD exposure affect mammary gland development during puberty. In AHR-responsive B6 mice, TCDD and DMBA can influence mRNA expression involved with mammary gland differentiation as well as genes involved with AHR ligand and estrogen metabolism; because D2 mice express the low-affinity AHR but are susceptible to diet-induced obesity, we further examined whether HFD-fed D2 mice exposed to TCDD and/or DMBA would be responsive to AHR-ligand–induced expression changes.

## Materials and Methods

### Chemicals

TCDD (99.9% pure; Ultra Scientific, North Kingstown, RI) and DMBA (98% pure; Sigma Chemical Company, St. Louis, MO) were obtained commercially. Both chemicals were dissolved in 95%/5% olive oil/toluene by volume (Sigma). DMBA and TCDD were dosed from 25 mg/mL and 500 ng/mL stock concentrations, respectively, corresponding to 2.4 μL DMBA solution/g mouse and 1.8–1.9 μL TCDD solution/g mouse.

### Mice and dosing

**Figure 1:**
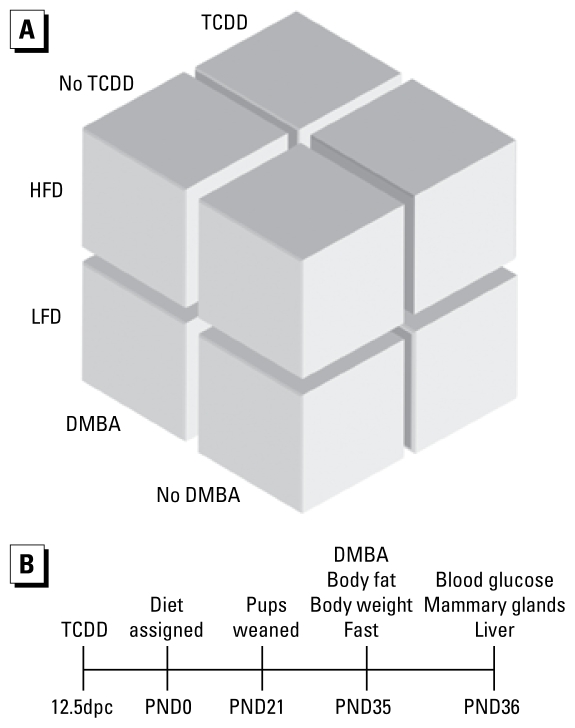
Schematic of treatment groups (*A*) and time line (*B*). Impregnated B6 and D2 nulliparous mice were treated with 1 μg/μL TCDD or 95%/5% olive oil/toluene (vehicle) at 12.5dpc. Dams received HFD or LFD at parturition, and pups were weaned onto the same diets.

B6 and D2 nulliparous female mice (Jackson Laboratory, Bar Harbor, ME) were mated with B6 and D2 males, respectively, and dosed with 1 μg/kg TCDD or vehicle control by oral gavage at 12.5 days postcoitus (dpc), corresponding to the time when fetal mammary fat pads are developing (*n* = 24 dams per strain and treatment group). On postnatal day (PND) 0, dams were changed from 5058 chow (Purina, St. Louis, MO) to an HFD (45% total kilocalories from fat and 35% total kilocalories from carbohydrate; D12451, Research Diets, New Brunswick, NJ; *n* = 12 dams) or a matched control low-fat diet (LFD; 10% total kilocalories from fat and 70% kilocalories from carbohydrate; D12450B, Research Diets; *n* = 12 dams) (Figure 1). Diets had the same percentage of protein, with fat differences being achieved through increased maltodextrin and lard and decreased cornstarch and sucrose in HFD compared with LFD (400, 1,598, 291, and 691 kcal vs. 140, 180, 1,260, and 1,400 kcal, respectively). The LFD has fat levels that fall into the range often found in standard rodent chows. Mice had *ad libitum* access to feed and water. Female pups were weaned at PND21, ending any lactational exposure to TCDD but continuing their respective HFD or LFD exposures. Female D2 offspring were dosed with 60 mg/kg DMBA (*n* = 12 litters) or vehicle (*n* = 12 litters) by oral gavage on PND35, when developing pubertal mammary glands are known to be sensitive to DMBA exposure. Mice were euthanized 24 hr later by carbon dioxide asphyxiation. All animal experiments were performed humanely using protocols to alleviate suffering and were approved by the University of North Carolina at Chapel Hill Institutional Animal Care and Use Committee and were performed in a vivarium accredited by the Association for Assessment and Accreditation of Laboratory Animal Care. Because survival of B6 offspring was greatly reduced in the HFD group treated with TCDD, B6 offspring were not analyzed further.

### Metabolic end points

On PND0, body weights of D2 progeny were assessed by weighing the entire litter and determining the average pup weight. Individual D2 body weights were measured at PNDs 4, 7, 10, 14, 18, 21, and 35. On PND35, D2 percent body fat was measured with a Lunar PIXImus dual-energy X-ray absorptiometry scanner (GE Lunar Corp., Madison, WI) using isoflurane anesthesia. Blood glucose levels were measured from tail blood on PND36 using a FreeStyle blood glucose kit (Abbott Laboratories, Abbott Park, IL) after a 24-hr fast.

### Histology

Inguinal mammary glands from D2 progeny were fixed and stained with carmine alum at PND36 to detect terminal end buds and branch elongation according to published methods (Fenton et al. 2002).

### Molecular analysis

At PND36, median liver lobes and inguinal mammary glands of D2 mice were dissected and homogenized, and RNA was extracted. We used the High-Capacity cDNA Archive Kit (Applied Biosystems, Foster City, CA) to generate cDNA for polymerase chain reaction (PCR) analysis. Real-time PCR was performed to assess relative transcript levels of *Cyp1a1*, *Cyp1a2*, *Cyp1b1*, *Areg* (amphiregulin), *Ereg* (epiregulin), *Egf (*epidermal growth factor), *Rac1* (Ras related C3 botulinum substrate 1), *Tgfb* (transforming growth factor beta), *Bax* (BCL-2 associate X protein), *Ccnd1* (cyclin d1), *Mki67* (antigen identified by monoclonal antibody Ki 67), *Igf1* (insulin-like growth factor 1), *Fgf2* (fibroblast growth factor 2), *Ahr*, *Egfr* (epidermal growth factor receptor), and *Esr1* (estrogen receptor, alpha) using Assays-on-Demand (Applied Biosystems), with *Gusb* (glucuronidase, beta) and *Actb* (actin, beta) as endogenous controls in hepatic and mammary tissues, respectively. Endogenous controls were selected based on serial titration performance and lack of treatment-specific expression variance.

### Statistical analysis

All analyses were performed using SAS, version 9.1.3 (SAS Institute Inc., Cary, NC). The litter median of female progeny traits was used as the unit of TCDD analysis to control for potential bias within litters due to maternal effects, although the result trends were identical when analyzing data for each pup individually. Three D2 litters (LFD + vehicle + vehicle, LFD + vehicle + DMBA, and HFD + TCDD + DMBA) had no females. Because of this imbalance in the design, Student’s *t*-test was not appropriate. Instead, we used the more conservative generalized linear model (Proc GLM; SAS Institute Inc.) to evaluate the effect of TCDD, DMBA, and diet on phenotypes (body weight, percent body fat, fasting blood glucose, branch elongation, fat pad length, number of terminal end buds, size of terminal end buds, and fold change) (Livak and Schmittgen 2001) of *Cyp1a1*, *Cyp1a2*, *Cyp1b1*, *Areg*, *Ereg*, *Egf*, *Rac1*, *Tgfb*, *Bax*, *Ccnd1*, *Mki67*, *Igf1*, *Fgf2*, *Ahr*, *Egfr*, and *Esr1* levels. All Proc GLM analyses modeled phenotypes with additive main effects (diet, TCDD, DMBA), along with all two-way interactions and the three-way interaction of the main effects. Any significant interactions were explored with stratified analyses. Using the LSMEANS option of Proc GLM, the multivariate geometric means were determined to be significantly different at unadjusted *p* < 0.05 across a limited number of *a priori* contrasts. We took this conservative analysis approach because of the small sample size and the imbalance between some contrasts (e.g., fewer exposed to DMBA than to vehicle).

## Results

### Variable progeny survival caused by maternal TCDD exposure

**Figure 2:**
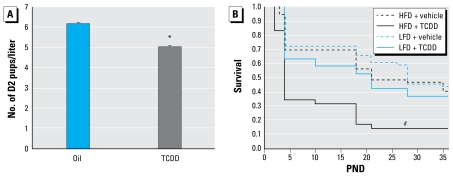
Treatment with TCDD or vehicle at 12.5dpc reduced D2 litter size and B6 early-life survival. (*A* ) TCDD (*n* = 13 litters) reduced D2 litter size compared with oil vehicle (*n* = 14 litters; mean + SE). (*B*) B6 survival (*n* = 37 litters) expressed as the fraction of pups present at birth; 1.0 corresponds to 100% survival. *p < 0.05. ^#^*p* < 0.0001.

Effects of TCDD on survival have been well characterized in adult mice. To examine the sensitivity to maternal TCDD exposure, we used TCDD doses that are < 200- and < 2,600-fold than the adult half-maximal lethal dose (LD_50_) of B6 and D2 mice, respectively (Chapman and Schiller 1985). The two mouse strains appeared to have different periods of sensitivity to maternal TCDD; D2 mice were more sensitive to transplacental TCDD exposure, whereas B6 mice were more sensitive to lactational TCDD exposure. At PND0, B6 litter size was not affected by maternal TCDD exposure. However, D2 females had a mean litter size of 4.7 when exposed to TCDD compared with a mean litter size of 6.3 pups when exposed to the vehicle (*p* < 0.05; Figure 2A). Lactational TCDD exposure did not reduce postnatal pup survival in D2 mice, and we observed no interaction between TCDD exposure and diet in D2 survival (data not shown). As opposed to their prenatal resistance to TCDD-induced mortality, B6 survival was diminished postnatally by maternal exposure to TCDD (*p* < 0.0001; Figure 2B). Most B6 pup mortality occurred during the period of lactation, and the decline in survivorship was most pronounced during the first post-natal week. Coexposure to perinatal TCDD and HFD significantly reduced B6 pup survival compared with all other treatment groups (*p* < 0.0001; Figure 2B); however, HFD did not appear to prolong the window in which TCDD reduced B6 pup survival. Because of low B6 survival, no additional end points were examined in B6 mice.

### Maternal TCDD exposure raises glucose levels in HFD-fed D2 progeny

**Figure 3:**
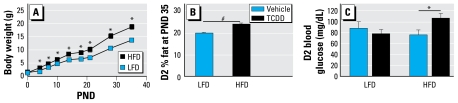
Diet and maternal TCDD exposure effects on body composition and fasting blood glucose. (*A*) HFD increased postnatal D2 body weights (mean ± SE; *n* = 27–31 at PNDs 0–26 for HFD, and *n* = 28 at PND35 for LFD). (*B*) HFD (*n* = 26 mice) increased percent fat at PND35 relative to LFD (mean ± SE; *n* = 28 mice). (*C* ) Fasting blood glucose was increased by HFD and maternal TCDD-treated (black bars; *n* = 5 litters) compared with HFD and maternal vehicle-treated (*n* = 6 litters) female progeny at PND36 (means ± SE). Because diet, but not TCDD, changed body weight and percent body fat, these analyses were done on individual D2 mice, with TCDD- and vehicle-treated D2 mice pooled within diet. *p < 0.05. ^#^*p* < 0.0001.

Body composition and glucose in D2 female progeny were analyzed for susceptibility to maternal TCDD exposure and diet. HFD increased postnatal D2 progeny growth compared with LFD as early as PND4 (*p* < 0.05; Figure 3A). Concordantly, pubertal D2 adiposity was 28.9% higher among D2 progeny fed HFD (25.6%) relative to those fed LFD (19.9%; *p* < 0.0001; Figure 3B). Although D2 progeny weigh more and have more body fat on an HFD than LFD, HFD did not significantly alter blood glucose compared with progeny fed LFD. Only in HFD-fed D2 progeny did maternal TCDD exposure heighten fasting blood glucose, by 59.3% over vehicle-treated D2 progeny (*p* < 0.05; Figure 3C). However, TCDD had no effect on either body mass or adiposity on D2 progeny fed either diet (data not shown).

### Pubertal mammary gland growth in D2 progeny is suppressed by maternal TCDD exposure

**Figure 4:**
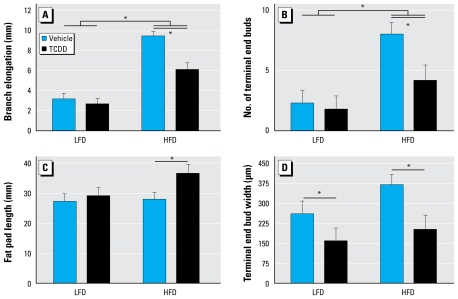
Alteration of mammary gland morphology by maternal TCDD exposure and HFD. Inguinal mammary whole mounts were made of glands removed at PND36 from progeny of vehicle- and TCDD-treated D2 mice fed HFD or LFD since weaning. Whole mounts were stained with carmine alum and measured for branch elongation (*A*), number of terminal end buds (*B*), fat pad length (*C*), and largest terminal end bud (*D*). Mean ± SE are shown for LFD groups: vehicle (*n* = 4 litters) and TCDD (*n* = 6 litters); and for HFD groups: vehicle (*n* = 6 litters) and TCDD (*n* = 5 litters). *p < 0.05 for diet effects and TCDD × diet effects.

Mammary glands from pubertal D2 female progeny were analyzed for susceptibility to maternal TCDD exposure and diet. D2 female mice maintained on HFD showed significant increases in branch elongation and number of terminal end buds compared with those maintained on LFD (*p* < 0.01; Figure 4A,B). However, relative to LFD, HFD was not associated with any changes in fat pad length or terminal end bud size in D2 female progeny (Figure 4C,D). TCDD primarily influenced mammary gland growth only in D2 progeny fed HFD, significantly increasing fat pad length (*p* < 0.05; Figure 4C) and significantly decreasing branch elongation (*p* < 0.05; Figure 4A), the number of terminal end buds (*p* < 0.05; Figure 4B), and the size of terminal end buds (*p* < 0.01; Figure 4D) relative to vehicle-treated D2 progeny fed HFD. In D2 progeny fed LFD, only terminal end bud size was significantly affected by maternal TCDD exposure (*p* < 0.05; Figure 4D). Similar to the effect of maternal TCDD, pubertal DMBA exposure reduced the number of terminal end buds to < 50% the number seen in vehicle-treated control D2 progeny (mean ± SE, 2.4 ± 0.8 and 5.0 ± 0.6, for *n* = 10 and 11 litters, respectively; *p*< 0.05; data not shown).

### Pubertal hepatic Cyp1 expression is elevated by maternal TCDD exposure

**Figure 5:**
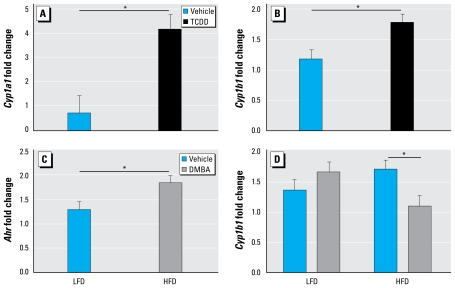
Maternal TCDD exposure and effect of diet on gene expression. Normalized message levels are represented as mean ± SE. (*A*) Induction of *Cyp1a1* was increased by TCDD exposure compared with vehicle (*n* = 11 and 10 litters, respectively). Measurements were pooled across diet and DMBA groups. (*B* ) Induction of *Cyp1b1* was increased by TCDD compared with vehicle (*n* = 11 and 10 litters, respectively). Measurements were pooled across diet and DMBA groups. (*C*) Induction of *Ahr* was increased by HFD relative to LFD (*n* = 11 and 10 litters, respectively). Measurements were pooled across TCDD and DMBA groups. (*D*) Induction of *Cyp1b1* by DMBA was decreased compared with vehicle in HFD-fed but not in LFD-fed D2 mice. LFD groups are vehicle (*n* = 5 litters) and DMBA (*n* = 5 litters); HFD groups are vehicle (*n* = 6 litters) and DMBA (*n* = 5 litters). *p < 0.05.

Maternal TCDD exposure increased hepatic *Cyp1a1* and *Cyp1b1* expression at puberty in D2 female progeny relative to vehicle exposure, independent of diet (*p* < 0.01; Figure 5A,B), whereas only HFD increased hepatic *Ahr* expression significantly compared with LFD at the same time point irrespective of TCDD exposure (*p* < 0.05; Figure 5C). Although DMBA and diet had no significant main additive effect on hepatic *Cyp1b1* expression, their interaction term was significant in the generalized linear model for hepatic *Cyp1b1* expression; when D2 female mice were maintained on HFD, DMBA induction of hepatic *Cyp1b1* expression was significantly reduced in progeny from the maternally exposed TCDD group relative to the maternal vehicle controls (*p* < 0.05; Figure 5D). When D2 female progeny were maintained on LFD, maternal TCDD exposure had no effect on hepatic *Cyp1b1* induction after DMBA exposure.

### Pubertal mammary gland fgf2 expression is elevated by LFD

Hepatic AHR activation indicates a liver response to xenobiotics, whereas mammary AHR appears to play a role in mammary gland development. Although interaction of AHR, EGFR, and ESR1 signaling in mammary glands has been suggested to be involved in both mammary morphogenesis and carcinogenesis (Buters et al. 1999; Ciarloni et al. 2007; Patel et al. 2006), we observed no effects of maternal TCDD exposure or diet on the expression of *Egfr*, or its ligand *Egf*, *Areg*, and *Ereg*, or of AHR activation indicators *Cyp1a1*, *Cyp1b1*, or *Esr1* in mammary glands of D2 female progeny (data not shown). Among additional growth factors involved in mammary morphogenesis (*Igf1*, *Rac1*, and *Fgf2*) only *Fgf2* gene expression was altered in mammary glands of D2 female progeny (Silberstein 2001). D2 mice fed LFD had 2.8-fold higher mammary *Fgf2* expression than did D2 mice HFD (*p* < 0.001; data not shown). All two-way and three-way interactions among diet, TCDD, and DMBA were statistically significant in *Fgf2* expression (*p* < 0.05). Markers of proliferation (*Mki67* and *Ccnd1*) and of apoptosis (*Bax*) were unaltered by all exposures in D2 mammary glands.

## Discussion

TCDD causes a broad range of toxic effects, yet its mechanisms are only partially understood and likely depend on many variables, such as dose, developmental stage of exposure, diet, and *Ahr* allele. The *Ahr* allele of D2 mice, like that of humans, codes for a receptor that weakly induces *CYP1A* (Moriguchi et al. 2003). In the present study, we treated D2 and B6 dams with 1 μg/kg TCDD, considered to be a low dose for mice (Poland and Glover 1980).

We demonstrate that B6 and D2 strains have different windows of reduced progeny survival in response to maternal TCDD exposure, with the severity being enhanced by HFD diet also in a strain-dependent manner. Although B6 litter size was unaffected by maternal TCDD exposure here and elsewhere (Vorderstrasse et al. 2004), postpartum B6 pup survival was greatly diminished by lactational exposure to TCDD and HFD. Postpartum D2 pups were relatively resistant to TCDD. However maternal TCDD exposure in D2 dams, as in *Ahr* null mice (Abbott et al. 1999), produced smaller litters. The strain-specific shift in timing of susceptibility to the effects of TCDD and HFD may be mediated by differences in the *Ahr* alleles between B6 and D2 mice.

The amount of TCDD that might accumulate in mice fed HFD is likely influenced by several factors. Increased adiposity slows TCDD elimination, extending its half-life (DeVito et al. 2003; Michalek and Tripathi 1999). Thus, upon TCDD exposure, larger fat depots of mice on HFD may sequester a higher cumulative dose of TCDD than in those on LFD (Hoppe and Carey 2007). Consequently, the higher target-organ dosage and slower elimination of TCDD in D2 progeny on HFD may exceed the minimum TCDD dose required to activate signaling. We focused on two variables potentially affecting susceptibility to this early-life TCDD exposure, DMBA and diet, because it is likely that an individual’s susceptibility to the effects of TCDD exposure is influenced by interactions with other environmental factors and body composition (Hakkak et al. 2007; Han et al. 2004; Thomsen et al. 2006). We found that maternal TCDD exposure and diet interact nonadditively to significantly alter both fasting blood glucose and mammary development of D2 female progeny at puberty.

The risk of insulin resistance, type II diabetes, and associated mortality has been linked to low levels of TCDD exposure in several epidemiologic studies (Consonni et al. 2008; Cranmer et al. 2000; Henriksen et al. 1997). Furthermore, a study of Vietnam veterans demonstrated an interaction between overweight and TCDD on type II diabetes risk (Fujiyoshi et al. 2006). Our analysis of fasting blood glucose levels after maternal TCDD exposure supports the reported interaction between adiposity and TCDD.

Similarly, our results are consistent with recent suggestions that a link between obesity and puberty may exist in girls (Slyper 2006); pubertal breast development is enhanced in overweight girls who eat diets high in poly-unsaturated fats (Britton et al. 2004). We found HFD caused precocious mammary development in female D2 mice. Yet the precocious gland development was substantially reduced by a combination of HFD and maternal TCDD exposure in the same female offspring that had elevated fasting blood glucose. Mammary growth from the combined TCDD and HFD exposure was equivalent to that seen in vehicle-treated D2 mice on LFD, where TCDD had less impact on gland morphology. TCDD may have influenced puberty mammary growth of HFD-fed mice more than that of LFD-fed mice because the reduced mammary development in LFD-fed mice may have masked detection of mammary growth hindrance by TCDD. These results are consistent with delayed adolescent breast development that is correlated with increased serum TCDD levels in peripubertal girls (Den Hond et al. 2002).

Gestational, but not lactational, TCDD exposure imprints on rat mammary gland epithelial morphology at least as early as PND4 and into adulthood (Fenton et al. 2002), which suggests that gestational exposure to TCDD had already begun modifying the offspring mammary bud by the time HFD was administered at birth. In humans, rats, and now mice, evidence supports that prepubertal TCDD exposures delays mammary differentiation.

*Ahr* transcription is down-regulated during adipogenesis (Shimba et al. 2003). The fact that basal hepatic *Ahr* transcript levels are higher in D2 female progeny maintained on HFD indicates that elevation of *Ahr* expression is likely a consequence of weight gain. AHR signaling can cross-talk with ESR1 and EGFR, two receptors that are up-regulated in overweight individuals (Lorincz and Sukumar 2006; Moral et al. 2003). AHR-mediated signaling pathways also interact with mammary morphogenesis during puberty (Howlin et al. 2006). Although several groups demonstrated a role for EGFR and its ligands both in mammary morphogenesis and in AHR-mediated TCDD activity (Ciarloni et al. 2007; Howlin et al. 2006; Patel et al. 2006), we found no maternal TCDD-, HFD-, or DMBA-mediated changes in expression of *Esr1*, *Egfr* genes or the EGFR ligand genes *Egf*, *Areg*, and *Ereg* in the mammary glands of D2 female progeny. Branching and ductal morphogenesis are regulated by *Tgfb*, *Rac1*, *Igf1*, and *Fgf2* (Ewald et al. 2008; Korah et al. 2007; Silberstein 2001). Of these, only *Fgf2*, which initiates and elongates ducts, was altered, suggesting that mammary glands of LFD-fed D2 mice were poised to have increased branching and elongation. Further, we found no evidence that proliferation and apoptosis influenced D2 mammary morphogenesis. Together, these findings suggest that mechanisms at a different developmental stage may be responsible for the effects of maternal TCDD and diet on mammary gland development.

In addition to cross talk with estrogen-mediated pathways, AHR mediates *Cyp1a1* and *Cyp1b1* induction by TCDD. Although neither *Cyp1a1* nor *Cyp1b1* transcripts were altered in the mammary gland after maternal TCDD exposure, we did observe a modest increase in hepatic *Cyp1a1* and *Cyp1b1* expression at puberty. Maternal TCDD could indirectly decrease estrogen by increasing its metabolism. In the liver and mammary glands, CYP1A1 and CYP1B1 generate the catechols 2-hydroxyestradiol and 4-hydroxyestradiol from estrogen, respectively (Tsuchiya et al. 2005). Recent evidence suggests that TCDD-stimulated production of these catechols is increased further in mice fed HFD (Zhu et al. 2008). If up-regulation of hepatic *Cyp1a1* and *Cyp1b1* transcripts by maternal TCDD in D2 female progeny translates to increased hepatic CYP1A1 and CYP1B1 protein, this should reduce mammary estrogen levels. Consequently, increased estrogen metabolism and less estrogen may be the mechanism that by which TCDD decreases mammary growth (Ciarloni et al. 2007; Howlin et al. 2006).

Because D2 mice have the *Ahr^d^* allele, they are considered nonresponsive to DMBA induction (Galvan et al. 2003, 2005). Thus, without maternal TCDD exposure, DMBA would not be metabolically activated in D2 female progeny (Chapman and Schiller 1985; Moriguchi et al. 2003). However, increased *Cyp1a1* and *Cyp1b1* transcripts caused by maternal TCDD exposure may lead to metabolic activation of DMBA in D2 mice (Chapman and Schiller 1985; Moriguchi et al. 2003). An important implication of these results is that maternal exposure to TCDD may increase susceptibility to DMBA-induced mammary carcinogenesis in mice typically having no susceptibility to DMBA. Because human AHR has similar activity as AHR^d^ of D2 mice (Moriguchi et al. 2003), this TCDD-increased susceptibility to DMBA may extend to humans. Thus, the potential interaction of maternal or low-level TCDD exposure, pubertal PAH exposure, and HFD on the risk of breast cancer incidence in humans should be investigated.

## Conclusions

DMBA and TCDD represent PAHs and PHAHs, respectively, that are ubiquitous chemical classes in the environment that frequently occur as mixtures in human and environ mental samples. Because of the prevalence of TCDD exposure and elevated adiposity in modern society, most people have some TCDD tissue burden. Women that have been exposed to TCDD can expose their children to TCDD through maternofetal transfer and/or breast-feeding, further increasing the risk of exposure to environmental mediators of breast cancer during key developmental windows of susceptibility. Our data from D2 mice, with similar AHR activity as humans (Moriguchi et al. 2003), suggest that increased adiposity may increase susceptibility to the effects of aromatic hydrocarbons among sub-populations perceived to have minimal health risk of such exposures. Low-dose TCDD exposure can alter pubertal mammary growth under distinct environmental and genetic contexts, which may alter risk to cancer-causing exposures. Our data suggest that greater focus needs to be placed on modeling all aspects of people in modern society to accurately reflect potential health effects of chemical exposures.
