# Errata

**Published:** 2009-10

In the article by La Merrill et al. [Environ Health Perspect 117:1414–1419 (2009)], the keys in Figure 3B and Figure 5C should have been in Figure 3C and Figure 5D, respectively. The corrected figures are provided below.

*EHP* apologizes for the errors.

## Figures and Tables

**Figure 3:**
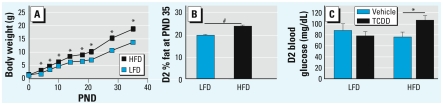
Diet and maternal TCDD exposure effects on body composition and fasting blood glucose. (*A*) HFD increased postnatal D2 body weights (mean ± SE; *n* = 27–31 at PNDs 0–26 for HFD, and *n* = 28 at PND35 for LFD). (*B*) HFD (*n* = 26 mice) increased percent fat at PND35 relative to LFD (mean ± SE; *n* = 28 mice). (*C*) Fasting blood glucose was increased by HFD and maternal TCDD-treated (*n* = 5 litters) compared with HFD and maternal vehicle-treated (*n* = 6 litters) female progeny at PND36 (mean ± SE). Because diet, but not TCDD, changed body weight and percent body fat, these analyses were done on individual D2 mice, with TCDD- and vehicle-treated D2 mice pooled within diet. *p < 0.05. ^#^*p* < 0.0001.

**Figure 5:**
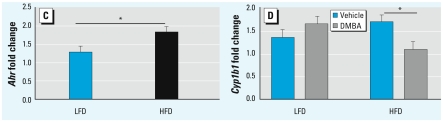
Maternal TCDD exposure and effect of diet on gene expression. Normalized message levels are represented as mean ± SE. (*C*) Induction of *Ahr* was increased by HFD relative to LFD (*n* = 11 and 10 litters, respectively). Measurements were pooled across TCDD and DMBA groups. (*D*) Induction of *Cyp1b1* by DMBA was decreased compared with vehicle in HFD-fed but not in LFD-fed D2 mice. LFD groups are vehicle (*n* = 5 litters) and DMBA (*n* = 5 litters); HFD groups are vehicle (*n* = 6 litters) and DMBA (*n* = 5 litters). *p < 0.05.

